# Liver transplantation versus surgical resection for HCC meeting the Milan criteria: A propensity score analysis: Retraction

**DOI:** 10.1097/MD.0000000000050362

**Published:** 2026-08-14

**Authors:** Jun-Yi Shen, Chuan Li, Tian-Fu Wen, Lv-Nan Yan, Bo Li, Wen-Tao Wang, Jia-Yin Yang, Ming-Qing Xu, Tholakkara Nazar Highness

The article, “Liver transplantation versus surgical resection for HCC meeting the Milan criteria: A propensity score analysis”,^[1]^ which appears in Volume 95, Issue 52 of *Medicine*, is being retracted due to insufficient evidence that the study did not use organs or data derived from executed prisoners. The authors were notified of this retraction.
